# Shift in Immune Parameters After Repeated Exposure to Nanoplastics in the Marine Bivalve *Mytilus*

**DOI:** 10.3389/fimmu.2020.00426

**Published:** 2020-04-15

**Authors:** Manon Auguste, Teresa Balbi, Caterina Ciacci, Barbara Canonico, Stefano Papa, Alessio Borello, Luigi Vezzulli, Laura Canesi

**Affiliations:** ^1^Department of Earth, Environment and Life Sciences (DISTAV), University of Genoa, Genoa, Italy; ^2^Department of Biomolecular Sciences (DIBS), University of Urbino, Urbino, Italy

**Keywords:** mussel, innate immunity, amino modified polystyrene, nanoplastics, immune training

## Abstract

Bivalves are widespread in coastal environments subjected to a wide range of environmental fluctuations: however, the rapidly occurring changes due to several anthropogenic factors can represent a significant threat to bivalve immunity. The mussel *Mytilus* spp. has extremely powerful immune defenses toward different potential pathogens and contaminant stressors. In particular, the mussel immune system represents a significant target for different types of nanoparticles (NPs), including amino-modified nanopolystyrene (PS-NH_2_) as a model of nanoplastics. In this work, the effects of repeated exposure to PS-NH_2_ on immune responses of *Mytilus galloprovincialis* were investigated after a first exposure (10 μg/L; 24 h), followed by a resting period (72-h depuration) and a second exposure (10 μg/L; 24 h). Functional parameters were measured in hemocytes, serum, and whole hemolymph samples. In hemocytes, transcription of selected genes involved in proliferation/apoptosis and immune response was evaluated by qPCR. First exposure to PS-NH_2_ significantly affected hemocyte mitochondrial and lysosomal parameters, serum lysozyme activity, and transcription of proliferation/apoptosis markers; significant upregulation of extrapallial protein precursor (EPp) and downregulation of lysozyme and mytilin B were observed. The results of functional hemocyte parameters indicate the occurrence of stress conditions that did not however result in changes in the overall bactericidal activity. After the second exposure, a shift in hemocyte subpopulations, together with reestablishment of basal functional parameters and of proliferation/apoptotic markers, was observed. Moreover, hemolymph bactericidal activity, as well as transcription of five out of six immune-related genes, all codifying for secreted proteins, was significantly increased. The results indicate an overall shift in immune parameters that may act as compensatory mechanisms to maintain immune homeostasis after a second encounter with PS-NH_2_.

## Introduction

Invertebrates represent more than 95% of animal diversity and are found in virtually any ecosystem, and the different species rely on its innate immune system to adapt and survive in its ecological niche. The mechanisms involved in “immune specificity” (sophisticated recognition systems for a wide variety of nonself material), as well as in “immune training/priming” (the capacity to mount a faster and more effective response upon reexposure to a stimulus), are therefore central to the capacity of invertebrates to survive in diverse environments (1–5). However, the rapid environmental changes induced by several anthropogenic factors can represent a significant threat to invertebrate immune defenses. This in particular applies to marine species, which encounter challenges associated with climate changes such as increased water temperature that may favor the growth of some pathogens (6), as well as pollution caused by a number of emerging contaminants, including nanoparticles (NPs) and plastic debris (microplastics and nanoplastics) (7, 8).

Bivalve mollusks (mussels, oysters, and clams) are widespread in coastal environments characterized by a wide range of environmental fluctuations. In bivalves, both cellular and humoral components of the immune system cooperate in the process of recognition and elimination of microbial and other nonself particles [reviewed in (8–10)]. Among bivalves, the mussel *Mytilus* spp. is particularly resistant to infection; they are able to cope with a large variety of potential pathogens, as well as contaminants. Taking advantage of the robust immune defenses of mussels, they have been employed as a model organism for studying the effects of different types of NPs (11–14).

Nanoplastics can be derived from fragmentation of microplastics and larger plastic debris (15–17). Amino-modified nanopolystyrene (PS-NH_2_) has been recently used as a model to study the effects of nanoplastics on marine invertebrates (18–23). The effects of PS-NH_2_ (50 nm) have been investigated on *Mytilus galloprovincialis* hemocytes *in vitro* (24 and references quoted therein). The results showed lysosomal stress and activation of immune parameters [lysozyme release, extracellular reactive oxygen species (ROS), and NO production]. Moreover, the formation of a stable biomolecular corona around PS-NH_2_ was identified in hemolymph serum (HS), whose unique component was represented by the extrapallial protein precursor (EPp), an immune-related, cation binding protein (24). The results underlined that the *in vitro* immunomodulatory properties of PS-NH_2_ were mediated by specific interactions with both humoral and cellular components of the mussel immune system.

In this work, the *in vivo* effects of PS-NH_2_ on the immune function of *M. galloprovincialis* were investigated. In particular, we evaluated the impact of nanoplastics on immune parameters after an acute exposure event to PS-NH_2_ (10 μg/L, 24 h; Expo1), the possible recovery after 72-h depuration (Resting), and the response to a second acute exposure (10 μg/L, 24 h; Expo2). Controls (unexposed mussels) were run in parallel. At each time point, several functional parameters were measured in hemocytes, serum, and whole hemolymph from exposed and control mussels. In hemocytes, transcription of genes related to proliferation and apoptosis, as well as a set of immune-related genes, was evaluated by qPCR.

## Materials and Methods

### Characterization of PS-NH_2_

Primary characterization of 50-nm nonfluorescent amino polystyrene NPs PS-NH_2_, purchased from Bangs Laboratories Inc. (Fishers, IN, USA), and analysis of their behavior in different aqueous media were carried out by a combination of analytical techniques as previously described (18, 24, 25). Average size, polydispersity index, and zeta potential of PS-NH_2_ suspensions (50 μg/L) in Milli-Q water, artificial seawater (ASW), and *Mytilus* hemolymph serum (HS) were evaluated by dynamic light scattering (DLS) (18, 24, 25). Since agglomeration and surface charge in different media were shown to play a key role in determining the interactions of this type of PS-NH_2_ with mussel hemocytes, these results are summarized in Table S1.

### Animals and Treatments

Mussels (*M. galloprovincialis* Lam.), 4–5 cm long, purchased from an aquaculture farm (Arborea, OR, Italy) in July 2018, were transferred to the laboratory and acclimatized for 24 h in static tanks containing aerated ASW, pH 7.9–8.1, 36 ppt salinity (1 L per animal), at 16 ± 1°C.

Stock suspension of PS-NH_2_ (25 mg/ml in water) was suitably diluted in Milli-Q water, quickly vortexed but not sonicated, and immediately spiked in the tanks in order to reach the final desired concentration of 10 μg/L per mussel (nominal concentration level).

**Figure 1:**
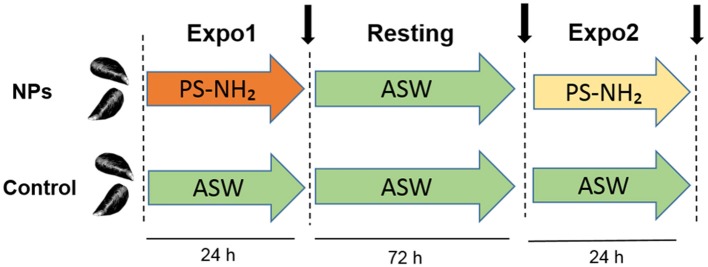
Schematic representation of the *in vivo* experiment on *Mytilus galloprovincialis* upon repeated exposure to PS-NH_2_ used in the present study. First exposure (Expo1): PS-NH_2_, 10 μg/L, 24 h; resting period: clean ASW, 72 h; and second exposure (Expo2): PS-NH_2_, 10 μg/L, 24 h. A control group of unexposed mussels was kept in clean ASW for the whole experiment time. For both conditions (control and PS-NH_2_-exposed mussels), sampling times are indicated (black arrows). Four independent experiments were performed (*n* = 4).

Mussels were first exposed to PS-NH_2_ (Expo1) at 10 μg/L for 24 h, followed by depuration in clean ASW for 72 h (Resting) and by a second exposure to PS-NH_2_ (10 μg/L for 24 h) (Expo 2). A parallel group of control (untreated) mussels was kept in clean ASW throughout the exposure time (see Figure 1 for details of the experimental setup). Seawater was changed daily. Animals were not fed during the experiments. At each time point (Expo1, Resting, and Expo2), hemolymph was extracted from the posterior adductor muscle of five mussels (from both control and exposed conditions), filtered through sterile gauze, and pooled in tubes at 16°C. Four independent experiments were performed (*n* = 4). Aliquots of whole hemolymph (from 50 to 200 μl, depending on the assay) were utilized for determination of different parameters. The remaining hemolymph was centrifuged at 100 × *g* for 10 min at 4°C, and the resulting supernatant was utilized for determination of serum lysozyme activity. The hemocyte pellet was resuspended in TRIzol reagent (Sigma, Milan, Italy) and stored at −80°C for gene expression analysis. All measurements were performed in triplicate.

### Hemocyte Counts

Flow cytometry (FC) was utilized to determine total hemocyte counts (THCs) and various cell types in mussel hemolymph from control and PS-NH_2_-exposed mussels in different experimental conditions, as previously described (26). Aliquots (50 μl) from the fresh hemocyte suspensions were added to 250 μl of PBS-NaCl (2 mM KH_2_HPO_4_, 10 mM Na_2_HPO_4_, 3 mM KCl, and 500 mM NaCl in distilled water, pH 7.4). Samples were analyzed by flow cytometry (FACSCalibur, BD Becton Dickinson, San Jose, CA, USA). Data acquisition and analysis were performed with the BD CellQuest software using the parameters of relative size (FSC) and granularity (SSC). Counting beads (DakoCytoCount™) were added in a volume of 50 μl to each tube. Five gates were set up to identify cell subpopulations, as well as spermatozoa, cell debris, and aggregates, which were not considered for further analysis. A representative 2D plot of control samples showing the three hemocyte subpopulations [R1: hyalinocytes (HY), R2: small granulocytes (SG), and R3: large granulocytes (LG)] is reported in Figure S1. Hemocyte viability was checked by propidium iodide (PI) staining as previously described (26), indicating >95% cell viability in samples from all experimental conditions (not shown).

### Evaluation of Hemocyte Functional Parameters

Lysosomal membrane stability (LMS) was evaluated by the neutral red retention time (NRRT) assay as previously described (27–29). Hemocyte monolayers on glass slides were incubated with 20 μl of neutral red (NR) solution (final concentration 40 μg/ml from a stock solution of NR 40 mg/ml in DMSO); after 15 min, excess dye was washed out and 20 μl of ASW was added. Every 15 min, slides were examined under an optical microscope, and the percentage of cells showing loss of the dye from lysosomes in each field was evaluated. For each time point, 10 fields were randomly observed, each containing 8–10 cells. The end point of the assay was defined as the time at which 50% of the cells showed signs of lysosomal leaking (the cytosol becoming red and the cells being rounded). In control mussels, no significant changes in LMS were observed throughout the experiment, with average high NRRT values of >120 min. Data (*n* = 4) are expressed as percentage of control values.

For confocal laser scanning microscopy (CLSM) analyses, hemocytes were fixed with paraformaldehyde at 4% for 10 min, washed two times for 2 min with TBS (0.05 M Tris-HCl buffer, pH 7.8), and permeabilized with 0.05% NP-40 (Nonidet-40) for 10 min as previously described (25, 30, 31). Mitochondrial membrane potential (MMP, Δψ_m_) was evaluated by the fluorescent dye tetramethylrhodamine ethyl ester perchlorate (TMRE). TMRE is a quantitative marker for the maintenance of the MMP, and it is accumulated within the mitochondrial matrix in accordance to the Nernst equation. TMRE exclusively stains the mitochondria and is not retained in cells upon collapse of the Δψ_m_. Hemocytes were incubated with 40 nM TMRE for 10 min and observed by confocal microscopy.

Dynamic changes and functions of the lysosomes were evaluated in hemocytes loaded with 125 nM of LysoSensor™ Green DND-189 for 45 min. The LysoSensor™ dye accumulates inside acidic vesicles and exhibits an increase in fluorescence intensity which is proportional to acidification (32).

Fluorescence of TMRE (excitation 568 nm, emission 590–630 nm) and LysoSensor™ Green DND-189 (excitation 443 nm, emission 505 nm) was detected using a Leica TCS SP5 confocal setup mounted on a Leica DMI6000 CS inverted microscope (Leica Microsystems, Heidelberg, Germany) using a 63 × 1.4 oil objective (HCX PL APO 63.0-1.40 OIL UV). Images were analyzed by the Leica Application Suite Advanced Fluorescence (LASAF) and ImageJ Software (Wayne Rasband, Bethesda, MA). TMRE and LysoSensor fluorescence intensities were measured as integrated fluorescence density (arbitrary units) per cell area in at least 12 different fields of each sample. Data (*n* = 4) are reported as percentage of control values.

### Serum Lysozyme Activity

Lysozyme activity in aliquots of hemolymph serum was determined spectrophotometrically at 450 nm utilizing *Micrococcus lysodeikticus* as previously described (29). Hen egg white (HEW) Lyso was used as a concentration reference, and lysozyme activity was expressed as HEW Lyso equivalents (U/ml/mg protein). Protein content was determined according to the bicinchoninic acid (BCA) method, using bovine serum albumin (BSA) as a standard. In control mussels, no significant changes in serum lysozyme activity were observed throughout the experiment, with overall average values of 50 ± 6 U/ml/mg of protein. Data (*n* = 4) are expressed as percentage of control values.

### Bacterial Cultures and Evaluation of Bactericidal Activity of Whole Hemolymph Samples

The sensitivity of *Vibrio aestuarianus* 01/032 to the bactericidal activity of mussel hemolymph was evaluated *in vitro* as previously described (33, 34). *V. aestuarianus* 01/032 was cultured in Zobell medium at 20°C under static conditions; after overnight growth, cells were harvested by centrifugation (4,500 × *g*, 10 min), washed three times with phosphate-buffered saline (PBS-NaCl: 0.1 M KH_2_PO_4_, 0.1 M K_2_HPO_4_, and 0.15 M NaCl, pH 7.2–7.4), and resuspended to obtain a concentration about 10^9^ CFU/ml (determined spectrophotometrically as Abs_600_ = 1).

Aliquots (1 ml) of whole hemolymph were incubated with a bacterial suspension of *V. aestuarianus* 01/032 containing 1 × 10^9^ CFU/ml, diluted in order to obtain a nominal concentration of 4 × 10^7^ CFU/ml, at 16°C for different periods of time. Triplicate preparations were made for each sampling time. Immediately after the inoculum (*T* = 0) and after 60 and 90 min of incubation, aliquots (0.1 ml) of hemolymph samples were placed in a tube containing 9.9 ml of ASW supplemented with 0.05% Triton X-100 and vortexed for 10 s to lyse the hemocytes. Tenfold serial dilutions in ASW of the lysate were plated onto Luria-Bertani (LB) agar 3% NaCl. After overnight incubation at 24°C, the number of colony-forming units (CFUs) was determined. Percentages of killing were compared with values obtained at zero time (*n* = 4). The number of CFUs in control samples never exceeded 0.1% of those of exposed samples.

### RNA Extraction and qPCR

Total RNA was extracted from hemocytes obtained from each condition (*n* = 4) using TRIzol reagent (Sigma, Milan, Italy) following the manufacturer's protocol. RNA concentration and quality were verified using the Qubit RNA assay (Thermo Fisher, Milan, Italy) and by electrophoresis using a 1.5% agarose gel under denaturing conditions. A first-strand cDNA for each sample was synthesized from 1 μg of total RNA (29). Gene transcription was evaluated in four independent RNA samples. Primer pairs employed for qPCR analysis were used as reported in previous studies (Table S2). qPCRs were performed in triplicate in a final volume of 15 μl containing 7.5 μl iTaq universal master mix with ROX (Bio-Rad Laboratories, Milan, Italy), 5 μl diluted cDNA, and 0.3 μM specific primers (Table S2). A control lacking a cDNA template (no-template) was included in the qPCR analysis to determine the specificity of target cDNA amplification. Amplifications were performed in a CFX96™ Real-Time PCR System (Bio-Rad Italy, Segrate, Milan) using a standard “fast mode” thermal protocol. For each target mRNA, melting curves were utilized to verify the specificity of the amplified products and the absence of artifacts. Relative quantification of each mRNA transcript was calculated by the comparative C_T_ method (35). Expression of the genes of interest was normalized using the expression levels of EF-α1 as a reference gene, and the normalized expression was then reported as relative quantity of mRNA (relative expression) with respect to control samples.

### Statistical Analysis

Data are the mean ± SD of four independent experiments (*n* = 4), with each assay performed in triplicate. Data of functional parameters and hemocyte counts were analyzed by two-way ANOVA followed by Tukey's test at 95% confidence intervals (*P* ≤ 0.05). For bactericidal activity and gene transcription, statistical differences were evaluated by the Mann–Whitney *U* test (*P* < 0.05). All statistical calculations were performed using the PRISM 7 GraphPad software.

## Results

### Flow Cytometry

**Figure 2:**
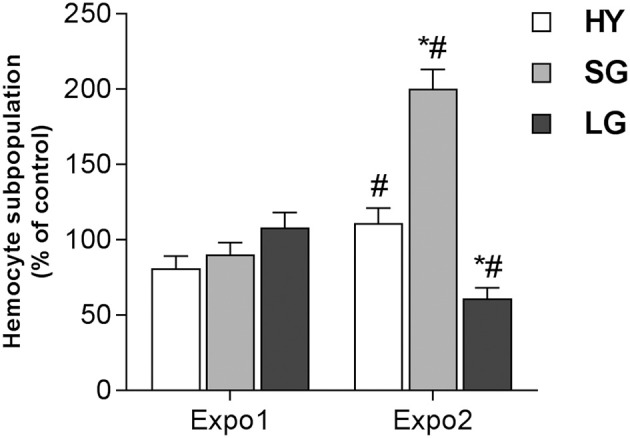
Effect of PS-NH_2_ on hemocyte subpopulations in *Mytilus galloprovincialis* hemolymph after first (Expo1) and second exposure (Expo2). HY = hyalinocytes, SG = small granulocytes, LG = large granulocytes. Data, expressed as percent values with respect to each control group, are the mean ± SD of four experiments. *all Expo. *versus* controls; ^#^Expo1. vs. Expo2. *P* ≤ 0.05 (ANOVA followed by Tukey's test).

In control hemolymph, THCs were about 1.2 ± 0.3 × 10^6^/ml. No significant changes were observed in control samples throughout the experiments; moreover, THCs were unaffected by either Expo1 or Expo2 to PS-NH_2_ (not shown). Based on particle size and granularity, three hemocyte subpopulations were identified, namely, HY, SG, and LG (Figure S1) as previously described (26, 36), with the sum of SG + LG accounting for more than 80% of total hemocytes in all experimental conditions. After Expo1 to PS-NH_2_, no significant changes in hemocyte subpopulations were observed (Figure 2). However, after Expo2, a large increase was observed in the percentage of SG (about +100% with respect to controls and Expo1; *P* ≤ 0.05); in contrast, the proportion of LG was significantly decreased (about −40% with respect to controls and Expo1). The percentage of HY was similar to controls but significantly higher (+30%; *P* ≤ 0.05) than that observed after Expo1 to PS-NH_2_.

### Measurement of Hemocyte Mitochondrial and Lysosomal Parameters by CLSM

**Figure 3:**
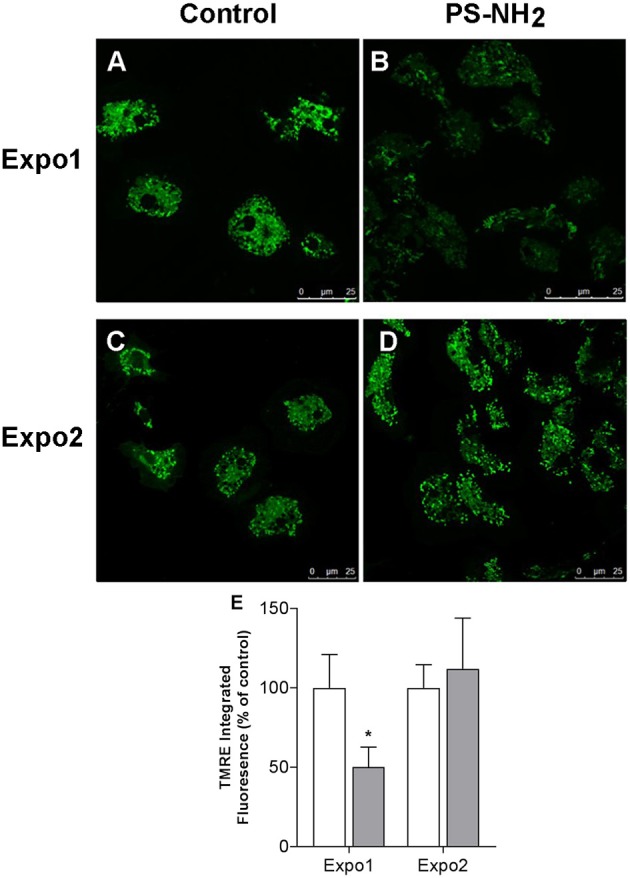
Confocal fluorescence microscopy: effects of exposure of *Mytilus galloprovincialis* to PS-NH_2_ on hemocyte mitochondrial membrane potential (ΔΨ_m_) evaluated by TMRE fluorescence. Hemocytes from control and exposed mussels were loaded with TMRE, and representative images are reported (568 excitation/590–630 emission). Expo1: **(A)** Control and **(B)** PS-NH_2_ exposed. Expo2: **(C)** Control and **(D)** PS-NH_2_ exposed. **(E)** Quantification of the TMRE fluorescence signal in hemocytes from control and PS-NH_2_-exposed mussels at each exposure. Data, expressed as percentage of integrated fluorescence density/cell area with respect to each control group, are the mean ± SD of four experiments. *P ≤ 0.05 (ANOVA followed by Tukey's test). Scale bars: 25 μm.

The effects of PS-NH_2_ on hemocyte mitochondria were evaluated by cell staining with TMRE, an indicator of MMP Δψ_m_, and representative CLSM images are reported in Figure 3, together with the quantification of the TMRE fluorescence signal. After Expo1, a net decrease in Δψ_m_ was observed (Figures 3A,B), as shown by the significant reduction in TMRE fluorescence (−50% with respect to control; *P* ≤ 0.05; Figure 3E). However, upon Expo2, no differences in fluorescence were recorded with respect to controls (Figures 3C,D).

**Figure 4:**
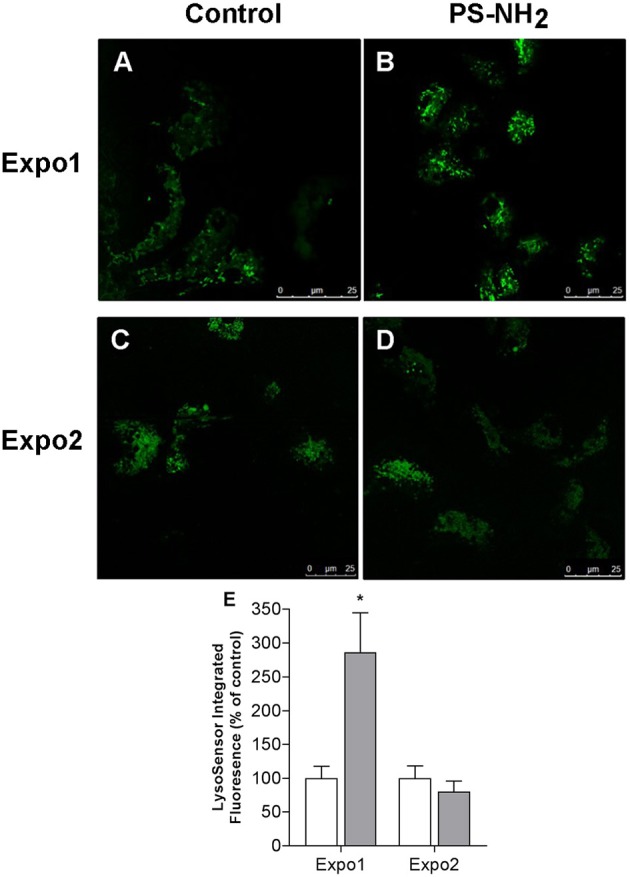
Confocal fluorescence microscopy: effects of exposure of *Mytilus galloprovincialis* to PS-NH_2_ on hemocyte lysosomal compartment evaluated by LysoSensor™ fluorescence and representative images (443 nm excitation/505 nm emission) are reported. Upper panel: hemocytes after Expo1, control **(A)** and PS-NH_2_ exposed **(B)**; lower panel: hemocytes after Expo2, control **(C)** and PS-NH_2_ exposed **(D)**. **(E)** Quantification of the LysoSensor fluorescence signal in hemocytes from control and PS-NH_2_-exposed mussels after each exposure. Data, expressed as percentage of integrated fluorescence density/cell area with respect to each control group, are the mean ± SD of four experiments. *P ≤ 0.05 (ANOVA followed by Tukey's test). Scale bars: 25 μm.

Similarly, the effect on hemocyte lysosomal compartments was evaluated using the fluorescent dye LysoSensor™, which becomes more fluorescent in acidic environments, and representative images are reported in Figure 4. Expo1 induced a clear increase in the LysoSensor signal (Figures 4A,B) (+186% with respect to controls; *P* ≤ 0.05; Figure 4E), whereas no effects were observed after Expo2 (Figures 4C,D).

### Functional Immune Parameters

**Figure 5:**
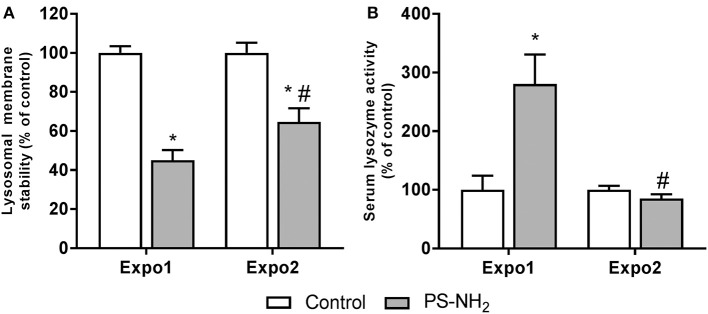
Effects of PS-NH_2_ exposure (10 μg/L) on *Mytilus galloprovincialis* hemocytes. **(A)** Lysosomal membrane stability (LMS); **(B)** serum lysozyme activity. Data, expressed as percent values with each respective control group, are the mean ± SD of four experiments. *all Expo. vs. controls; ^#^Expo1. vs. Expo.; *P* ≤ 0.05 (ANOVA followed by Tukey's test).

The effects of PS-NH_2_ exposure on hemocyte and hemolymph immune functional parameters were evaluated, and the results are reported in Figure 5. LMS was first evaluated as a functional marker of the lysosomal function related to cellular stress and immune response. Expo1 resulted in a significant drop in LMS (about −50% with respect to control, *P* ≤ 0.05). However, after Expo2, a smaller effect was observed (−30% with respect to control, *P* ≤ 0.05) (Figure 5A). Expo1 also induced a large increase in serum lysozyme activity (+150% with respect to controls, *P* ≤ 0.05) (Figure 5B), whereas no effects were observed after Expo2. In contrast, other immune parameters (phagocytic activity and extracellular ROS production) were not affected in any experimental condition (Figure S2). In order to assess possible recovery of functional parameters, hemocyte LMS, and Δψ_m_ and hemolymph lysozyme activity were evaluated after a 72-h resting period, as representative parameters of hemocytes and hemolymph serum, respectively. All parameters showed full recovery after depuration (Figure S3).

**Figure 6:**
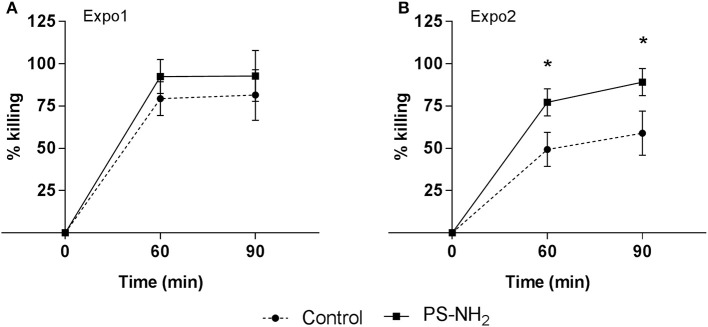
Effects of PS-NH_2_ exposure on *Mytilus galloprovincialis* hemolymph bactericidal activity. *In vitro* bactericidal activity toward *Vibrio aestuarianus* 01/032 of whole hemolymph samples from control (dotted line) and PS-NH_2_-exposed mussels (black line), **(A)** after Expo1 and **(B)** after Expo2. Hemolymph was incubated with *V. aestuarianus* for 60 and 90 min as described in section Materials and Methods. Percentages of killing were determined in comparison to values obtained at zero time. Data, expressed as percent values with each respective control group, are the mean ± SD of four experiments (*P < 0.05) (Mann–Whitney's *U* test).

The overall immune function was evaluated in whole hemolymph samples challenged *in vitro* with *V. aestuarianus* 01/032 for 60 and 90 min. Data, expressed as percentage of killing activity, are presented in Figure 6. In control samples of Expo1, bactericidal activity at 60 min (Figure 6A) was higher with respect to that of controls of Expo2 (Figure 6B), whereas similar values were observed at 90 min; however, all data fell within the range of killing of this vibrio strain by mussel hemocytes (34). The first exposure to PS-NH_2_ did not affect the bactericidal activity toward *V. aestuarianus* 01/032 with respect to controls at both times of incubation (Figure 6A). However, after Expo2, a significant increase was observed in samples from PS-NH_2_-exposed mussels at both 60 and 90 min (+51% and +56%, respectively, *P* ≤ 0.05) compared to control samples (Figure 6B). When the bactericidal activity of hemocytes alone (in the presence of ASW and absence of hemolymph serum) was evaluated, no killing of *V. aestuarianus* 01/032 was observed in any experimental condition as previously described (34).

### Effects on Hemocyte Gene Expression

**Figure 7:**
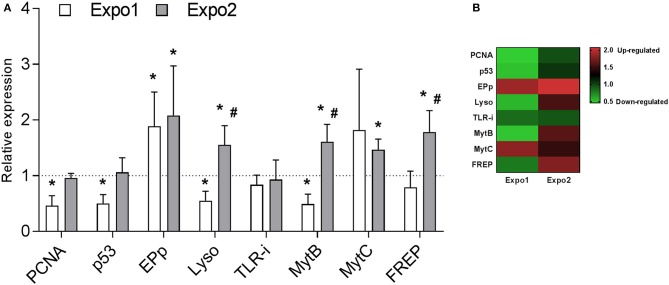
Effect of PS-NH_2_ exposure on gene transcription in *Mytilus galloprovincialis* hemocytes evaluated by qPCR. **(A)** Relative expression of proliferating cell nuclear antigen (PCNA), tumor suppression protein 53 (p53), extrapallial protein precursor (EPp), lysozyme (Lyso), Toll-like receptor i isoform (TLR-i), mytilin B (MytB), myticin B (MytC), and fibrinogen-related proteins (FREPs) after Expo1 (white bars) and Expo2 (gray bars). Data, reported as relative expression with respect to each control group, are the mean ± SD of four independent RNA samples. *all Expo. vs. controls; ^#^Expo1. vs. Expo2. *P* < 0.05 (Mann–Whitney's *U* test). **(B)** Heatmap of eight differentially expressed genes was generated in each sample.

Transcription of a set of selected genes involved in cell proliferation and apoptosis [proliferating cell nuclear antigen (PCNA) and tumor suppression protein 53 (p53), respectively] and in immune response [Extrapallial protein precursor (EPp), Lyso, Toll-like receptor i isoform (TLR-i), mytilin B (MytB), myticin B (MytC), and fibrinogen-related protein (FREP)] was evaluated by qPCR. Data on relative expression of each transcript (fold changes with respect to control) are reported in Figures 7A,B.

The results show that Expo1 induced a significant decrease in mRNA levels for both PCNA and p53 (about −50% with respect to controls, *P* ≤ 0.05). In contrast, after Expo2, transcription of both genes was similar in hemocytes from control and treated samples. Expo1 significantly affected transcription of three out of six immune-related genes (Figure 7A). The expression of EPp was upregulated (+88%, *P* ≤ 0.05), whereas that of Lyso and MytB was downregulated (−45% and −51%, respectively, *P* ≤ 0.05). After Expo2, a distinct gene expression pattern was observed. Five genes were upregulated with respect to controls: EPp (+107%, *P* ≤ 0.05), Lyso (+54%, *P* ≤ 0.05), the antimicrobial peptides (AMPs) MytB and MytC (+60 and +46%, respectively; *P* ≤ 0.05), and FREP (+78%, *P* ≤ 0.05). TLR-i expression was unaffected in either exposure condition. Data are summarized in the heatmap reported in Figure 7B.

## Discussion

Previous data showed that short-term *in vitro* exposure to PS-NH_2_ significantly affected immune parameters of *M. galloprovincialis* hemocytes (24, 25). In the present study, the *in vivo* effects of PS-NH_2_ were evaluated. The main aims of the work were (i) to gather information on the impact of acute (24 h) exposure to PS-NH_2_ on functional immune parameters; (ii) to investigate the responses induced by repeated exposure to PS-NH_2_ on the overall immune function; and (iii) to gain a first insight on the possible molecular mechanisms involved by evaluating the transcription of a set of selected genes.

The results show that Expo1 to PS-NH_2_ significantly affected functional parameters of circulating hemocytes in terms of MMP, lysosomal acidification, and membrane destabilization and also increased lysozyme release in the hemolymph, indicating degranulation (Figures 3–5). No changes in THCs and hemocyte subpopulations (Figure 2) or in hemocyte phagocytic activity and ROS production (Figure S2) were observed. However, transcription of PCNA and p53 was affected, suggesting modulation of proliferation/apoptotic processes (Figure 7). The results confirm previous *in vitro* data obtained with PS-NH_2_ (24, 25) and underline the occurrence of stress conditions in the hemocytes, which did not however result in significant changes in the overall hemolymph bactericidal activity. Accordingly, after 72-h depuration, key functional parameters in hemocytes and serum (lysosomal stability, MMP, and lysozyme activity, respectively) showed full recovery (Figure S3). This is in line with the observation that, in bivalve tissues, nanopolystyrene particles of similar size are rapidly depurated within three days (20).

Upon Expo2 to PS-NH_2_, a distinct pattern of responses was observed. Hemocyte MMP and lysosomal acidification, as well as serum lysozyme activity, were similar in control and exposed mussels (Figures 3, 4, 5B). Moreover, a significantly smaller lysosomal membrane destabilization in hemocytes was recorded with respect to that induced by Expo1 (Figure 5A), corresponding to minor cellular stress (37, 38). Although THCs were unaffected, a shift in hemocyte subpopulations was observed (Figure 2), with a decrease in LG, which represent the fully mature phagocytes (39, 40). This may be the result of massive cell degranulation indicated by the large increase of lysozyme release after Expo1. A parallel increase in the percentage of SG was detected (Figure 2): since hemocyte subpopulations represent the progressive maturation stages of a single cell type (39), this indicates a maturation process of granular phagocytic cells. Such a homeostatic process is also suggested by the complete recovery of mRNA levels of genes involved in proliferation/apoptosis in the whole hemocyte population. In particular, with regard to apoptotic processes, a decrease in mitochondrial membrane depolarization, which represents a pre-apoptotic signal, was observed only after Expo1, and not after resting or Expo2 (Figure S3); accordingly, FC data on annexin/PI staining did not show significant changes in different experimental conditions (not shown).

Although phagocytosis of PS-NH_2_ was not evaluated, preliminary FC data were obtained using fluorescently labeled PS-NH_2_ of similar size (blue PS-NH_2_, 45–55 nm, Sigma Aldrich). This type of NPs showed the same agglomeration in exposure media as nonfluorescent PS-NH_2_, as well as comparable effects on mussel immune parameters (data not shown). The results indicate that after Expo1, uptake of nanoplastics occurred in about 34% of total cells (with over 90% represented by granulocytes, SG + LG). In contrast, a much smaller uptake was observed after Expo2 (about 7%). However, these results were only indicative, due to the low fluorescence signal of the particles utilized (data not shown).

On the basis of these data, though not conclusive, it is likely that mussels are able to establish tolerance mechanisms in immune defenses upon repeated, acute exposure to nanoplastics; in this light, these results are in line with those recently obtained in *M. galloprovincialis* after repeated, long-term exposure to polyethylene microplastics (18 days' first exposure; 28 days' depuration; 18 days' second exposure) to simulate the temporal variability of microplastics concentrations (41). Whole-transcriptome profiling at the tissue level revealed that, despite the physiological impairment triggered by the first exposure to microplastics, after the second exposure a decrease of stress- and immune-related gene expression was observed, indicating the establishment of compensatory mechanisms (41). It was suggested that mussels may be able to establish a stress memory upon microplastics exposure.

However, the results of the present work also show that after Expo2 to nanoplastics, the bactericidal activity of whole hemolymph was significantly increased, demonstrating a stimulation of the overall immune capacity. When expression of immune-related genes was evaluated, a distinct pattern was observed after the first or second exposure to PS-NH_2_ (Figure 7). Such a shift was evident for three genes that, after Expo1, showed downregulation (Lyso and MytB) or no changes (FREP) but that were upregulated after Expo2. Moreover, transcription of EPp and MytC was generally upregulated in both exposure conditions. Expression of the TLR-i was not affected in any experimental condition. Interestingly, the five genes that were upregulated after Expo2 to PS-NH_2_ codify for hemocyte-secreted proteins: activation of the molecular machinery involved in the synthesis and release of immune effectors may partly explain the mechanisms underlying the stimulation of hemolymph bactericidal activity observed upon repeated exposure to nanoplastics. In fact, bactericidal activity of *V. aestuarianus* 01/032 could be observed only in the presence of hemolymph serum, indicating a key role for soluble components as previously described (34). However, due to the variety of secreted immune proteins, the exact components responsible for the increase in bactericidal activity induced by Expo2 cannot be identified. Some of them may participate in direct bacterial killing, others in bacterial recognition and binding. In particular, upregulation of EPp, also known as the MgC1q6 isoform, observed at both times of exposure, may represent a specific effect of this type of nanoplastics. EPp, the most abundant serum protein in *M. galloprovincialis*, is an acidic, histidine-rich, cation binding glycoprotein; it has a complex and anomalous N-glycan structure and contains a conserved C1q complement domain. Due to its peculiar composition, EPp is involved in multiple functions, from shell formation to immune response (42–44). This protein has been shown to play a key role in specific recognition of both selected bacterial strains and NP types. EPp promotes mannose-sensitive interactions between *Mytilus* hemocytes and different bacterial strains of *V. aestuarianus* and *Vibrio cholerae* expressing mannose-sensitive hemagglutinin (MSHA) and *Escherichia coli* MG1655, carrying type 1 fimbriae, leading to activation of the immune response (34, 45). Moreover, EPp represents the unique protein component of the stable biomolecular corona formed around PS-NH_2_ in mussel hemolymph serum, which mediates specific recognition of this NP type by hemocytes and consequent immune response *in vitro* (18, 46). The persistent upregulation of EPp mRNA levels induced by PS-NH_2_ at both exposure times may result in increased levels of the protein in the hemolymph. Due to the multiple roles of this protein, this would contribute to the formation of the specific EPp-PS-NH_2_ corona in the hemolymph, affecting the interactions of PS-NH_2_ with hemocytes and consequent responses. Moreover, since EPp acts as a specific opsonin toward *V. aestuarianus* 01/032, its upregulation may lead to increased bactericidal activity of whole hemolymph samples toward this strain. Overall, the results indicate that mussel hemocytes are able to mount a distinct and more efficient immune response upon repeated exposure to PS-NH_2_. However, more experimental data, including measurements of immune responses after *in vivo* infection with *V. aestuarianus* 01/032, as well as with other vibrios, are needed to support this hypothesis. Preliminary data were obtained in mussels subjected to Expo1 conditions as in the present work and then challenged *in vivo* for 24 h with different vibrios. The results indicate that pre-exposure to PS-NH_2_ increased the hemolymph bactericidal activity toward *V. aestuarianus* 01/032, but not toward *Vibrio tasmaniensis* LGP32 (not shown), suggesting a specific response to this vibrio strain.

The concept of innate immune memory is now fairly accepted due to accumulating evidence in literature (47, 48). Innate immune memory can be defined as the ability of the immune system to store or simply use the information on a previously encountered antigen or parasite upon a secondary exposure (1). Three main mechanisms have been identified: the first is called recall or trained response, expressed as potentiation (with parameters showing enhanced response/activity upon the second exposure); the second is represented by a sustained, unique response which corresponds to the maintenance of a high response between exposures; and the last is characterized by an immune shift, a change in the response observed between several exposures (2, 3). However, evidence for epigenetic reprograming of immune cells (i.e., histone acetylation/deacetylation and DNA methylation) leading to changes in gene expression, which represent the characteristic hallmark of immune training or memory, has not been provided yet in most invertebrate groups, including bivalve mollusks (2–4).

On the other hand, evidence for immune stimulation induced by repeated challenge with natural pathogens is available in clams and oysters (49–55). In the mussel *M. galloprovincialis*, increased bactericidal activity was observed after *in vivo* and subsequent *in vitro* challenge with *Vibrio anguillarum* (26). Recent transcriptomics data obtained in *M. galloprovincialis* hemocytes after repeated challenge with *Vibrio splendidus* demonstrated a shift from a pro-inflammatory response to an anti-inflammatory and probably regenerative phenotype, indicating the existence of a secondary immune response in mussels oriented to tolerate infection (56).

Induction of innate memory mechanisms by NPs has been recently suggested for human monocytes primed with gold NPs (57). With the knowledge that NPs are able to modulate and induce immune responses similarly as natural pathogens do, in bivalves, they might at least contribute to mount a faster and/or stronger response upon a second display. Overall, repeated exposure of mussels to PS-NH_2_ resulted in a shift in granular hemocyte subpopulations, together with reestablishment of basal functional parameters and expression of proliferation/apoptotic markers, stimulation of bactericidal activity, and upregulation of immune gene transcription. These data indicate that both tolerance and potentiation may represent compensatory mechanisms to maintain immune homeostasis after a second encounter with PS-NH_2_. Experiments are in progress to investigate this possibility in more detail.

Bivalves express a wide range of inducible immune-related genes codifying for extracellular recognition and effector proteins, including lectins, peptidoglycan recognition proteins, lipopolysaccharide and β1,3-glucan-binding proteins, FREPs, and AMPs (58). The sequencing of the *Mytilus* genome reveals a very complex organization with high heterozygosity, abundant repetitive sequences, and extreme intraspecific sequence diversity among individuals (58–61). This complex machinery would be responsible for the high capacity of mussels to cope with microbial infection and environmental stress.

The present study demonstrates that NPs differentially stimulate the immune responses of *Mytilus*, and this species could serve as a model to explore the impact of nanoplastics on marine invertebrates, that respresents a major environmental concern.

## Data Availability Statement

The raw data supporting the conclusions of this article will be made available by the authors, without undue reservation, to any qualified researcher.

## Ethics Statement

The Mediterranean mussel, *M. galloprovincialis*, is not considered an endangered or protected species in any international species catalog, including the CITES list (www.cites.org), and not included in the list of species regulated by EC Directive 2010/63/EU. Therefore, no specific authorization is required to work on mussel samples.

## Author Contributions

MA, TB, and LC conceived and designed the study. MA, TB, CC, BC, and AB performed the experiments. CC, SP, and LV wrote sections of the manuscript. MA, TB, and LC wrote the manuscript. All authors contributed to manuscript revision, read, and approved the submitted version.

### Conflict of Interest

The authors declare that the research was conducted in the absence of any commercial or financial relationships that could be construed as a potential conflict of interest.
