# Supplementary material for: Shift in Immune Parameters After Repeated Exposure to Nanoplastics in the Marine Bivalve Mytilus
Source: Front Immunol. 2020 Apr 15;11:426. doi: 10.3389/fimmu.2020.00426 (PMC7174705; doi:10.3389/fimmu.2020.00426)
Supplement: Supplementary file 1 [file Data_Sheet_1.docx]

Supplementary Material

# Supplementary Tables

**Table S1-** Physico-chemical characterization of PS-NH_2_ suspensions (50 µg/L) in different exposure media (for experimental details see Canesi et al., 2015, 2016; Balbi et al., 2017). Data on average size, polydispersity index and zeta-potential are reported in MilliQ water, artificial seawater (ASW) and in hemolymph serum (HS) (mean ± SD, n=4).

|  | **Z-average (nm)^1^** | **PDI^2^** | **ζ-potential (mV)^3^** |
| --- | --- | --- | --- |
| **MilliQ water** | 57 ± 2 | 0.07 ± 0.02 | + 42.8 ± 1 |
| **ASW** | 200 ± 6 | 0.3 ± 0.02 | + 14.2 ± 2 |
| **HS** | 186 ± 3 | 0.34 ± 0.05 | + 14.2 ± 1 |

^1^Determinedby Dynamic Light Scattering (DLS)

^2^Polydispersity Index (PDI), determined by DLS

^3^ζ = zeta potential, determined by DLS

**Table S2- Primer sequences and qPCR performances.**

| Gene | Primers | Amplicon size bp | Amplification  efficiency (%) | Accession number | References |
| --- | --- | --- | --- | --- | --- |
| *EF-* α*1* | 5’- CGTTTTGCTGTCCGAGACATG -3’  5’- CCACGCCTCACATCATTTCTTG -3’ | 135 | 99 | AB162021 | Ciocan et al., 2011 |
| *EPp* | 5’- TAAACTCTGGACACGCATACC -3’  5’- GAGTCCCTCTTGGTGCATATT -3’ | 100 | 100 | AY364453 | Balbi et al., 2016 |
| *Lysozyme* | 5’- ATGTGGAATCTGAAGGACTTGT -3’  5’- CCAGTATCCAATGGTGTTAGGG -3’ | 368 | 99 | AF334665 | Li et al., 2008 |
| *TLR-i* | 5’- AGGATGGCTTGAACTGGATT -3’  5’- AGTCGAGTAGGCTTTCTGTA -3’ | 110 | 100 | JX173690 | Toubiana et al., 2013 |
| *MytB* | 5’- GTTATTCTGGCTATCGCTCTTG -3’  5’- GTATAATGTCAAACAGAACGGGTC -3’ | 357 | 103 | AF162336 | Cellura et al., 2007 |
| *MytC* | 5’- CTTCTGTCTTCATTGCAGCA -3’  5’- ACTGGTGAGTGTAACGTGTGC -3’ | 290 | 104 | AF162335 | Cellura et al., 2007 |
| *FREP* | 5’- CCTGACAAATGCAACAGTGG -3’  5’- TGGCCGTTGTGATGTTCTAA -3’ | 389 | 102 | HQ236392.1 | Romero et al., 2011 |
| *PCNA* | 5’- CTGAGCTAGAGTTCTGTGCCA -3’  5’- AGTTTG GAG ACT CGGTTGTGA -3’ | 108 | 105 | LNJA010048824.1 | This study |
| *p53* | 5’- CAAACTTGCTAAATTTGTTGAAGA -3’  5’- TTGGTCCTCCTACACATGAC -3’ | 140 | 105 | DQ158079 | Dondero et al., 2006 |

EF1 = *M. galloprovincialis* Elongation factor-1α; EPp= *M. edulis* Extrapallial protein precursor; Lysozyme = *M. galloprovincialis* Lysozyme; TLR-i = *M. galloprovincialis* Toll-like receptor i; MytB = *M. galloprovincialis* antimicrobial peptide precursor mytilin B; MytC = *M. galloprovincialis* antimicrobial peptide precursor myticin B; FREP = *M. galloprovincialis* fibrinogen-related protein; PCNA = *M. galloprovincialis* proliferating cell nuclear antigen; p53 = *M. galloprovincialis* p53-tumor suppressor-like protein.

# Supplementary Figures

**Supplementary Figure 1**– Representative FC analysis of control hemocytes using size FSC and internal complexity SSC as discriminating parameters. The three subpopulations were identified on a linear FSC-SSC density plot according to their SSC and FSC ranges as hyalinocytes-HY (R1: low FSC and SSC),small granulocytes-SG (R2: medium SSC and FSC) and large granulocytes-LG (R3: high SSC and FSC).


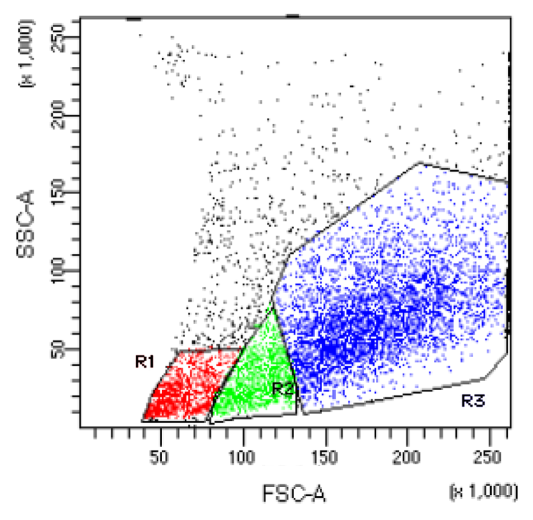


**Supplementary Figure 2-** Effects of PS-NH_2_ exposure on *M. galloprovincialis* hemocytes. **(A)** Phagocytic activity; **(B)** extracellular ROS production. Data are reported as mean (N=4) ± SD. Phagocytic activity was evaluated as uptake of Neutral Red-conjugated zymosan particles in hemocyte monolayers and extracellular generation of reactive species was measured by thereduction of cytochrome c, as previously described (Canesi et al., 2015).


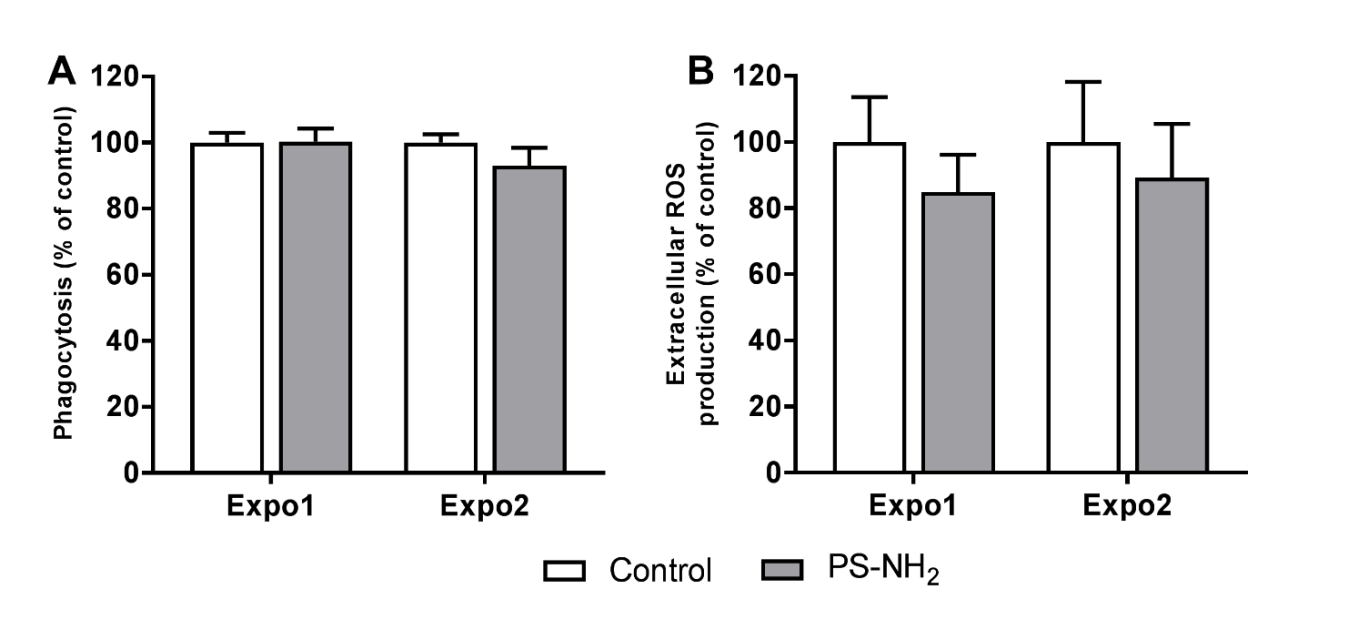


**Supplementary Figure 3-**Hemocyte and hemolymph parameters assessed at different steps along the whole experiment: Exposure 1 (Expo1), 72 h resting (Rest), Exposure 2 (Expo2). **(A)** Lysosomal membrane stability-LMS; **(B)** Mitochondrial membrane potential-TMRE; **(C) S**erum lysozyme activity. Data are reported as mean (N=4) ± SD. * = P ≤ 0.05, all exposures *vs* controls and Resting conditions; # = P ≤ 0.05, Rest and Expo 2 *vs* Expo 1 (ANOVA followed by Tukey's **test**).


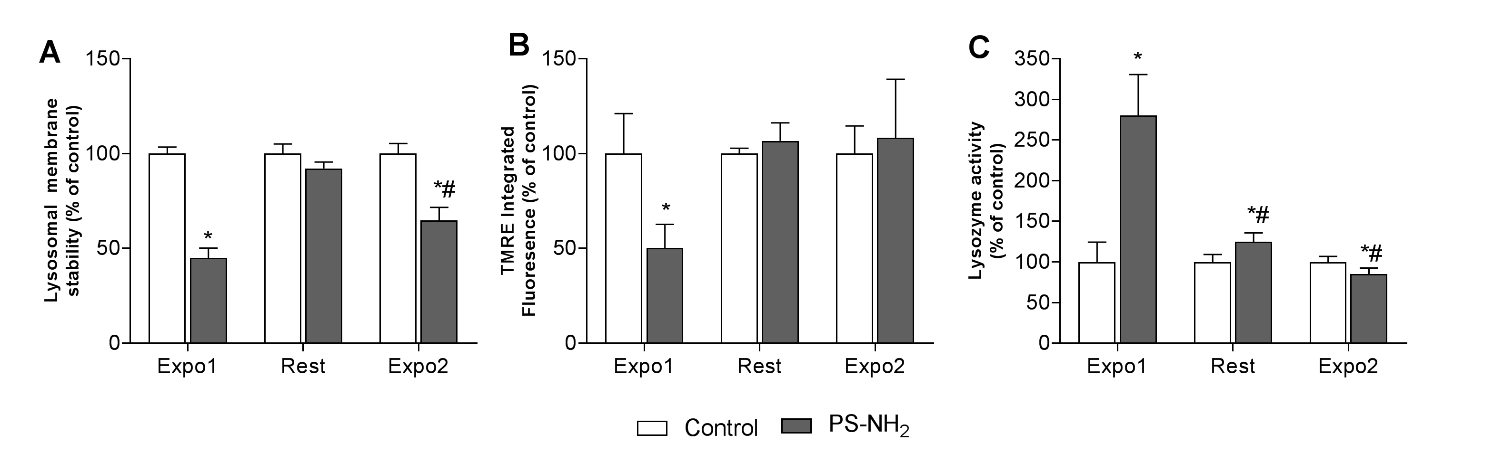


# References

Canesi, L., Ciacci, C., Fabbri, R., Balbi, T., Salis, A., Damonte, G., Cortese, K.,Caratto, V., Monopoli, M.P., Dawson, K.A., Bergami, E., Corsi, I., 2016. Interactions of cationic polystyrene nanoparticles with marine bivalve hemocytes in a physiological environment: Role of soluble hemolymph proteins. Environ. Res. 150, 73-81.

Canesi, L., Ciacci, C., Bergami, E., Monopoli, M.P.,Dawson, K.A.,Papa, S.,Canonico, B.,Corsi,I., 2015. Evidence for immunomodulation and apoptotic processes induced by cationic polystyrene nanoparticles in the hemocytes of the marine bivalve *Mytilus*. Mar. Environ. Res. 11, 34-40.

Balbi, T., Camisassi, G., Montagna, M., Fabbri, R., Franzellitti, S., Carbone, C., Dawson, K., Canesi, L., 2017. Impact of cationic polystyrene nanoparticles (PS-NH_2_) on early embryo development of *Mytilus galloprovincialis*: Effects on shell formation. Chemosphere, 186, 1-9.

Balbi, T., Franzellitti, S., Fabbri, R., Montagna, M., Fabbri, E., Canesi, L., 2016. Impact of bisphenol A (BPA) on early embryo development in the marine bivalve *Mytilus*: effects on gene transcription. Environ. Pollut. 218, 996-1004.

Ciocan, C.M., Cubero-Leon, E., Minier, C., Rotchell, J.M., 2011. Identification of reproduction-specific genes associated with maturation and estrogen exposure in a marine bivalve *Mytilus edulis*. PLoSOne 6, e22326.

Cellura, C., Toubiana, M., Parrinello, N., Roch, P., 2007. Specific expression of antimicrobial peptide and HSP70 genes in response to heat-shock and several bacterial challenges in mussels. Fish Shellfish Immunol. 22, 340–350. doi:10.1016/j.fsi.2006.06.007

Dondero, F., Piacentini, L., Banni, M., Rebelo, M., Burlando, B., Viarengo, A., 2005. Quantitative PCR analysis of two molluscan metallothionein genes unveils differential expression and regulation. Gene 345, 259–270. doi:10.1016/j.gene.2004.11.031

Li, H., Parisi, M.-G., Toubiana, M., Cammarata, M., Roch, P., 2008. Lysozyme gene expression and hemocytebehaviour in the Mediterranean mussel, *Mytilusgalloprovincialis*, after injection of various bacteria or temperature stresses. Fish Shellfish Immunol. 25, 143–152. https://doi.org/10.1016/j.fsi.2008.04.001

Romero, A., Dios, S., Poisa-Beiro, L., Costa, M.M., Posada, D.,Figueras, A.,Novoa, B., 2011. Individual sequence variability and functional activities of fibrinogen-related proteins (FREPs) in the Mediterranean mussel (*Mytilus galloprovincialis*) suggest ancient and complex immune recognition models in invertebrates. Dev. Comp. Immunol. 35, 334-344.

Toubiana, M., Gerdol, M., Rosani, U., Pallavicini, A., Venier, P., Roch, P., 2013. Toll-like receptors and MyD88 adaptors in *Mytilus*: Complete cds and gene expression levels. Dev. Comp. Immunol. 40, 158–166. doi:10.1016/j.dci.2013.02.006
